# Oral Microbial Species and Virulence Factors Associated with Oral Squamous Cell Carcinoma

**DOI:** 10.1007/s00248-020-01596-5

**Published:** 2020-11-06

**Authors:** Manolito G. Torralba, Gajender Aleti, Weizhong Li, Kelvin Jens Moncera, Yi-Han Lin, Yanbao Yu, Michal M. Masternak, Wojciech Golusinski, Pawel Golusinski, Katarzyna Lamperska, Anna Edlund, Marcelo Freire, Karen E. Nelson

**Affiliations:** 1 Department of Genomic Medicine, J. Craig Venter Institute, 4120 Capricorn Lane, La Jolla, CA 92037 USA; 2 Department of Genomic Medicine, J. Craig Venter Institute, 9605 Medical Center Drive Suite 150, Rockville, MD 20850 USA; 3 Burnett School of Biomedical Sciences, College of Medicine, University of Central Florida, 6900 Lake Nona Blvd, Central Florida Blvd, Orlando, FL 32827 USA; 4 Department of Head and Neck Surgery, Poznan University of Medical Sciences, The Greater Poland Cancer Centre, Garbary 15, 61-866 Poznan, Poland; 5 Department of Otolaryngology and Maxillofacial Surgery, University of Zielona Gora, Podgórna 50, 65-246 Zielona Góra, Poland; 6 Laboratory of Cancer Genetics, Greater Poland Cancer Centre, 15th Garbary Street, room 5025, 61-866 Poznan, Poland

**Keywords:** Oral cancer, Sequencing, Microbiome, Oral microbiome, Oral squamous cell carcinoma, *Fusobacterium*

## Abstract

The human microbiome has been the focus of numerous research efforts to elucidate the pathogenesis of human diseases including cancer. Oral cancer mortality is high when compared with other cancers, as diagnosis often occurs during late stages. Its prevalence has increased in the USA over the past decade and accounts for over 40,000 new cancer patients each year. Additionally, oral cancer pathogenesis is not fully understood and is likely multifactorial. To unravel the relationships that are associated with the oral microbiome and their virulence factors, we used 16S rDNA and metagenomic sequencing to characterize the microbial composition and functional content in oral squamous cell carcinoma (OSCC) tumor tissue, non-tumor tissue, and saliva from 18 OSCC patients. Results indicate a higher number of bacteria belonging to the Fusobacteria, Bacteroidetes, and Firmicutes phyla associated with tumor tissue when compared with all other sample types. Additionally, saliva metaproteomics revealed a significant increase of *Prevotella* in five OSCC subjects, while *Corynebacterium* was mostly associated with ten healthy subjects. Lastly, we determined that there are adhesion and virulence factors associated with *Streptococcus gordonii* as well as from known oral pathogens belonging to the *Fusobacterium* genera found mostly in OSCC tissues. From these results, we propose that not only will the methods utilized in this study drastically improve OSCC diagnostics, but the organisms and specific virulence factors from the phyla detected in tumor tissue may be excellent biomarkers for characterizing disease progression.

**Electronic supplementary material:**

The online version of this article (10.1007/s00248-020-01596-5) contains supplementary material, which is available to authorized users.

## Introduction

Oral cancer (OC) includes any cancer that affects head and neck tissues including mouth cancer, tongue cancer, tonsil cancer, and throat cancer. Over 40,000 individuals in the USA are diagnosed with a form of OC each year, and more than 481,000 new patients are diagnosed annually worldwide [1, 2]. The etiology of OC is multifactorial, and the incidence is increasing. Based on the available evidence, etiological factors include tobacco usage, excess consumption of alcohol, betel quid usage, HIV, HPV, periodontal diseases, exposure to sunlight, and ethnicity [3–5]. For most countries, 50% of individuals with cancers of the tongue, oral cavity, or oropharynx present 5-year survival rates. In case of late diagnosis, survival rates drop to 15%. Though there have been limited improvements in OC treatment, the mortality rate remains high at 43%, and the 5-year survival rate is discouraging at 56%. These bleak mortality numbers, however, are in part due to a majority of the diagnoses occurring during the late stages of OC development [1, 6]. The common occurrence of these late diagnoses can be attributed to a lack of consistent OC prevention programs, the inability to identify early-stage OC in asymptomatic individuals, and the lack of consistent biological markers to use for diagnostic purposes [7–9]. Fortunately, according to the Surveillance, Epidemiology, and End Results (SEER) database and the National Cancer Institute (NCI), OC has an 80–90% relative survival rate when found early compared with the overall population [10]. In oral squamous cell carcinoma (OSCC), the 5-year survival rate of 80% is reduced to 20–40% if the cancer is diagnosed at later stages (T3 and T4). One major factor behind the mechanism of high mortality in OSCC is the lack of early-stage molecular markers. Fortunately, to improve early diagnosis, the use of biofilm and saliva for the detection of OC has started to come to fruition with promising results [5, 11–15]. However, since there are no clear and consistent microbial biomarkers associated with OSCC progression, it is likely that using multi-omic approaches such as molecular characterization of OSCC as outlined in our study can lead to improved diagnostics, prevent late cancer stage detection, as well as increasing the potential for novel therapeutics.

Advancements in genomics approaches have allowed researchers to characterize entire microbial communities to understand complex interactions between the host and its microbiome. Conserved genomic markers such as the 16S rDNA gene (16S) have been used to characterize microbial diversity in these communities to further understand how their interactions with the host may contribute to health or pathogenesis. Although previous studies that utilized culture-based approaches, PCR, and sequencing of the oral microbiome revealed abundances and diversity of pathogens, there is a lack of multi-omic approaches to map the bacterial virulence associated with the taxonomic changes that are likely shaping disease phenotypes. Additional genomics approaches including metagenomics and transcriptome sequencing can also be used to understand these interactions. Efforts such as the Human Microbiome Project (HMP) have pioneered these approaches, which continue to have a significant impact on increasing our understanding of host–microbiome interactions [16]. These efforts, when applied to focus on specific interactions between the host and their microbial communities, are critical to understanding the pathology of complex diseases such as cancer. OC is one cancer where these approaches can provide novel insight into understanding pathology and most importantly into improving diagnostic approaches.

Previous studies have shown various associations between specific microorganisms and cancer [4, 17–19]. Most notable are the associations of *Helicobacter pylori* and gastric cancers as well as human papillomavirus (HPV) and cervical, genital, mouth, and throat cancers [20–24]. Studies have shown an association of various species of *Streptococcus* with OSCC using Sanger sequencing, Roche 454 pyrosequencing, and DGGE profiling in oral swabs and saliva from healthy and OSCC patients [5, 25–27]. These results, however, are perplexing since there are species of *Streptococcus* that are classified as commensal bacteria such as *Streptococcus salivarius*, *S. sanguinis*, *S. oralis*, and *S. mitis*, given their association with healthy oral microbial communities [28, 29] contrary to other species such as *S. mutans*, *S. intermedius*, and *S. constellatus*, which are known to be associated with the development of caries, dental plaque, and periodontal disease [28, 30, 31]. Additionally, when analyzing broader taxonomic differences, at the genus and phylum level, results from these studies are often inconsistent. For example, Schmidt and colleagues used 16S rRNA amplicon sequencing to show a reduction in *Streptococcus* and *Rothia* in OC, whereas Guerro-Preston et al. showed the opposite [32, 33]. Wang et al. also used 16S rRNA amplicon sequencing and showed little variation in alpha diversity between tumor and non-tumor tissues along with demonstrating the low abundance of *Actinomyces* and high abundance of *Parvimonas* in tumor tissues while having no mention of *Streptococcus* abundance in their study [34]. Lastly, Schmidt et al. (2014) showed decreased relative abundance of *Streptococcus* and *Rothia* in oral swabs from tumor lesions when compared with contralateral swab controls and healthy individuals [32]. The inconsistencies in existing studies clearly suggest a new approach to understanding the microbial community as it relates to OSCC pathology is necessary.

Though there have been a number of attempts to characterize the microbial communities associated with OSCC [5, 13, 35], these associations have yet to be fully determined. In the study presented here, we validated our findings by using high throughput sequencing on tumor and contralateral non-tumor oral tissues in conjunction with proteomics approaches on saliva derived from OSCC patients. More importantly, analyses of tissues allowed for a thorough assessment of the microbial community particularly bacteria such as *Fusobacterium* that have adherent and invasive characteristics and have been implicated in colorectal cancer [15, 36, 37] and leukoplakia [38]. More recent transcriptomics analysis by Yost et al. (2018) supports the role that *Fusobacterium* may have in the development of OSCC, showing a higher number of fusobacterial transcripts in tumor adjacent samples [39]. In the present study, we compare 16S rRNA amplicon and metagenomics sequences of tumor and healthy tissues of OSCC patients. From this dataset, we have been able to characterize candidate virulence factors from known oral pathogens that appear to be exclusive to tumor tissues. Additionally, we have profiled the host proteome of salivary OSCC samples [14] and further evaluated the metaproteomic composition of these samples to validate our sequencing data. To the best of our knowledge, this study is the first oral microbiome study that utilizes deep Illumina sequencing in conjunction with proteomics approaches to link microbial community structure and function in oral tissues with OSCC.

## Methods

### Sample Collection

Samples including non-tumor tissue (*n* = 18), tumor tissue (*n* = 18), and saliva (*n* = 18) were collected from OSCC patients enrolled in the study at the Greater Poland Cancer Centre (GPCC) Poznan, Poland. Of the samples collected, seven tumors were excised from the palatine tonsil, three were excised from the throat, three were excised from the bottom of the oral cavity, and five were excised from the tongue. Matched contralateral non-tumor tissues were also collected. Additional saliva samples (*n* = 5) collected from other members of the same cohort were randomly selected for proteomics analysis to further support data generated from the first 18 participants. Saliva samples from the original 18 samples were exhausted after DNA extraction and prior to proteomics analysis resulting in the use of saliva samples from other members of the cohort. Inclusion criteria included patients who only had surgery as a primary treatment. All patients with the previous diagnosis of any cancer, history of cancer treatment, including radiation and chemotherapy, and HPV-positive tumors were excluded from this study. Tumor tissue (TT) and contralateral non-tumor tissue (NT) was surgically excised from each patient in the outpatient clinic of the head and neck surgery department at the GPCC. Saliva was collected from OSCC patients following standard operating procedures for saliva sample collection as outlined by the Human Microbiome Project (HMP) [40]. Relevant clinical metadata including age, gender, and tumor location was collected. All samples were flash-frozen in liquid nitrogen and stored in ultra-low freezers (− 80 °C) after collection. Samples were later transported on dry ice to the J. Craig Venter Institute (JCVI). All authors declare that all methods in this study followed the protocol approved by the Institutional Review Board of Poznan University of Medical Sciences in Poznan, Poland, under IRB# 412/18. All experiments were performed in accordance with relevant guidelines and regulations. Informed consent for participation in the study was obtained from all patients included in the study.

### DNA Extraction, 16S rRNA PCR, and Sequencing

DNA from 18 patient TT, NT, saliva, and blank negative sample controls was extracted using lysozyme and proteinase K enzymatic digests followed by phenol-chloroform-isoamyl alcohol extraction and ethanol precipitation. We utilized 20–30 mm diameter of tissue for each extraction along with blank extraction negative controls. Extracted DNA was quantified using Sybr Gold (Thermo Fisher, Waltham, MA) via TECAN assay (Tecan Systems, Inc., San Jose, CA) in preparation for 16S PCR. Samples, extraction negative controls, and no template controls were amplified using Platinum Taq DNA polymerase (Thermo Fisher) and barcode and adaptor-ligated 16S primers [41] targeting the V4 region of the 16S rRNA gene fragment [42, 43]. Cycling conditions included 95 °C initial denaturing step for 5 min, followed by 35 cycles of 95 °C for 30 s, 57 °C for 30 s, and 72 °C for 30 s, followed by a final extension step of 72 °C for 5 min and 4 °C hold. Amplicons were then purified using the QIAquick PCR purification kit (Qiagen Inc., Hilden, Germany) in order to remove residual dNTPs and primer dimers according to the manufacturer’s specification. Purified amplicons were then quantified via TECAN, normalized, and pooled in preparation for 16S sequencing using the MiSeq platform (Illumina, La Jolla, CA) with V2 chemistry 500 cycles, 2 × 250 bp dual index format using standard manufacturer’s specifications. Sequences generated from 16S amplicons are available in Genbank under NCBI project PRJNA666891 stored as SRP ID SRP286018 in the Short Read Archive (SRA). 

### Metagenomics Sequencing

We selected genomic DNA (gDNA) extracted from healthy and tumor tissue from eight patients based on tumor location type. Eight TT samples were selected according to their anatomical location: palatine tonsil (*n* = 2), bottom of the oral cavity (*n* = 2), throat (*n* = 2), and tongue (*n* = 2), in addition to contralateral NT samples from the same patients to serve as controls. TT and NT gDNA extracts were enriched for microbial DNA using the NEB Microbiome Enrichment kit using standard manufacturer’s specifications (New England Biolabs, Ipswich, MA). Metagenomic libraries were prepared from 100 ng of enriched microbial DNA using the NEBNext Ultra II DNA library preparation kit for Illumina sequencing (New England Biolabs). Libraries were prepared by targeting 300–400 bp fragments for adapter ligation and amplification. Six PCR cycles were utilized to generate fragment libraries in preparation for sequencing. PCR reactions were purified according to specifications provided by NEB. Libraries were then checked for quality control using High Sensitivity DNA Lab on a Chip (Agilent Technologies, Santa Clara, CA) and the KAPA Library quantification kit according to manufacturer’s specification. The libraries that passed quality control were then normalized and pooled in preparation for sequencing on the Illumina NextSeq platform. Libraries were sequenced using the NextSeq 500, Mid Output kit, 300 cycles 2 × 150 bp, using manufacturer’s specifications (Illumina Inc.). Sequences that did not pass quality filtering were discarded, while high-quality sequences were binned according to Illumina indexes utilized during library preparation. Metagenomic sequences were deposited into Genbank under NCBI project PRJNA666891 stored as SRP ID SRP286018. 

### Illumina 16S Sequence Processing and Data Analysis

Unprocessed sequences were demultiplexed using CASAVA v1.8.2 (Illumina Inc., La Jolla, CA) to produce individual .fastq files for each pair of corresponding dual indexes. The DNA sequences were processed to ensure that only sequences with quality scores >Q30 were applied to the mothur pipeline in addition to utilizing stringent settings to ensure that no barcode mismatches were permitted among the demultiplexed reads [30]. Additional sequence filtering was used by applying the screen.seqs function of mothur to remove all sequences shorter than 220 bp [44]. Other QC steps were implemented, and the sequences were aligned against the SILVA database version 132 [45] to confirm the orientation of noise-filtered sequences along with ensuring the correct positioning of the reads with respect to which variable regions were amplified and sequenced. The sequences passing QC were then checked for chimeras using chimera slayer in mothur [44]. Sequences were classified taxonomically using the RDP classifier, and hits matching mitochondria, chloroplast, archaea, unknown, and eukaryote were eliminated to avoid noise on the data [46]. Sequence reads were then clustered at various taxonomic levels including 97% rDNA sequence similarity (OTU), genus, and phylum level. OTU table was imported in R and rarefied with a minimum library size of 4611 reads using vegan package version 2.5 [47]. Significant differences between sample groups were tested using pairwise permutation multivariate analysis of variance (PERMANOVA) with 999 permutations using ADONIS within RVAideMemoire package. Taxonomic differences between sample types were calculated using the SIMPER function in vegan. Core microbiome was computed using the script compute_core_microbiome.py in Qiime [48]. OTUs present in at least 65% of samples in each group were considered as core OTUs. Functional profiles were predicted based on 16S rRNA data using the Tax4fun software package and SILVA database [49]. Random Forest analysis was applied to the 16S rRNA data using machine learning methods developed by Leo Breiman to identify the most significant microbial features of our dataset. Features with a minimum prevalence of 10% across samples were included, and those with > 0.005 accuracy were considered significant. Data was further transformed to centered log ratio (CLR) before applying the Random Forest classification algorithm.

### Functional Potential of Non-Tumor and Tumor Tissue–Associated Metagenomes

Sequences were filtered for low-quality reads and human sequence contaminants using KneadDATA version 0.5.4 (available at http://huttenhower.sph.harvard.edu/kneaddata). Reads were scanned with a four-base wide sliding window and trimmed when the average base Phred score dropped below 20. Further, reads that were shorter than 70 nucleotides were discarded. Human genome assembly version hg38 (available at https://www.ncbi.nlm.nih.gov/grc/human) was used as a reference for removal of human contaminant sequences from the sequencing data. Taxonomy profiling of metagenomic sequences after quality filtering and human reads removal was performed by mapping the reads to a JCVI in-house microbial reference genome database with Centrifuge [50]. The reference database includes 27,115 representative genomes covering bacteria, archaea, viruses, fungi, and microbial eukaryotes species selected from NCBI RefSeq genomes. Species with relative abundance at least 1e−4 reported by Centrifuge were kept in this study.

Mapping the biosynthetic gene cluster (BGC) abundances for functional annotations was performed as described previously [51]. Briefly, both forward and reverse quality filtered FASTQ reads were aligned using BWA-MEM [52], Burrows–Wheeler aligner-maximum exact matches, with default parameters against the oral BGC database encompassing 4915 BGCs from oral bacteria [51]. For each gene, we summarized the counts of mapped reads from SAM alignment files using custom Perl script. We then applied the awk command to merge the individual count files generated from several mapping events. Count files were normalized using the DESeq2 pipeline [53] in R version 3.4.0 (https://www.r-project.org/), and size factors were determined using the median ratio method “poscounts,” which compensates for geometric means in samples where all genes have a value of zero. For significance testing, we performed 2-way ANOVA (Fisher’s least significant difference) on the DESeq normalized count data since negative binomial model–based Wald significance test (implemented by DESeq2) is more stringent for low count genes.

A virulence factor database was constructed by downloading 4,071,128 open reading frames (ORFs) from 490 well-curated oral bacterial genomes, available via the expanded Human Oral Microbiome Database (*e*HOMD) (available at http://www.homd.org/ftp/HOMD_prokka_genomes/). By using BLASTN, these ORFs were searched against the virulence factor database (VFDB) version 2019 (available at http://www.mgc.ac.cn/cgi-bin/VFs/v5/main.cgi), containing 3204 experimentally verified virulence genes of pathogenic bacteria. Closest matching amino acid sequences were selected with a minimum sequence identity cutoff at 75% and with a coverage of 75%. We adapted the above read mapping approach to evaluate relative abundances of these virulence genes in TT-, NT-, and saliva-associated metagenomes [54]. In order to quantify the abundances of antibiotic resistance genes (ARGs) in NT and TT metagenomes, ORFs from oral bacterial genomes were BLASTN searched against the antimicrobial resistance database MEGARes [55], containing a collection of 4000 curated ARG sequences. Subsequently, we mapped metagenome reads against potential oral ARGs to evaluate the abundances and performed enrichment analysis using 2-way ANOVA on the DESeq2 normalized counts as described above. In addition, for in-depth analysis of *Fusobacterium* functional potential, we aligned metagenomes against the 132-putative virulence and antibiotic resistance gene sequences collected mainly from multiple strains of *F. nucleatum* and *F. polymorphum* genomes. We applied the same read mapping approach as described above to analyze the differential enrichment of genes in tissue samples from healthy and cancer sites. In order to distinguish disease-associated genes, we further clustered DESeq2 normalized gene abundances based on Pearson’s correlation coefficient.

### Metaproteomics Analysis of OSCC Saliva

Five OSCC saliva samples from the same cohort were randomly selected along with five control saliva samples from a non-OSCC cohort and were subjected to metaproteomics analyses as previously described [14]. In brief, whole saliva samples were first thawed on ice and an aliquot of similar protein amount (20 ~ 30 μg) from each sample after SDS PAGE estimation [56] was digested using suspension trapping (STrap) approach with in-house (JCVI)-made glass fiber filters (Whatman GF/F, 0.7 μm). The protein digests were then analyzed by a nanoLC and Q-Exactive MS system (ThermoFisher Scientific, Waltham, MA) following a 150 min gradient on a 19-cm reversed phase column, particle size 3.0 μm, ReproSil-Pur C18-AQ media (Dr. Maisch GmbH, Ammerbuch-Entringen Germany). Global protein identifications were obtained by searching tandem mass spectra against a combined database of the Human Oral Microbiome Database (http://www.homd.org/, 1,079,626 protein sequences) and the UniProt human proteins (https://www.uniprot.org/proteomes/UP000005640, 20,349 reviewed sequences) using the Proteome Discoverer software (version 2.2, Thermo Scientific) and Sequest-HT algorithm [57].

## Results

### Sequencing Data Statistics

Post sequencing trimming and quality control of raw 16S sequences resulted in 772,366 sequence reads, with each sample averaging approximately 14,300 reads. Sequence reads are available at the Short Read Archive (SRA) under accession number (*will update once available*). OTU table was rarefied (normalized) to an even depth of 4611 reads. All samples generated an adequate number of reads for 16S analysis. Negative controls generated minimal sequence data and were not included in our analysis. Metagenomic sequencing on the resulted in approximately 101 M sequence reads, including both human and bacterial, with each sample averaging 6.36 M sequence reads after trimming and QC. After human sequences were removed, 5–16% (~ 390,000 to ~ 1.5 M) of the reads belonged to bacteria. Five metagenomic samples: P05-TT, P19-NT, P19-TT, P20-NT, and P20-TT, did not produce adequate sequence coverage and were omitted from our metagenomic analysis.

### Microbial Composition Associated with Tumor Tissue, Non-Tumor Tissue, and Saliva

**Fig. 1:**
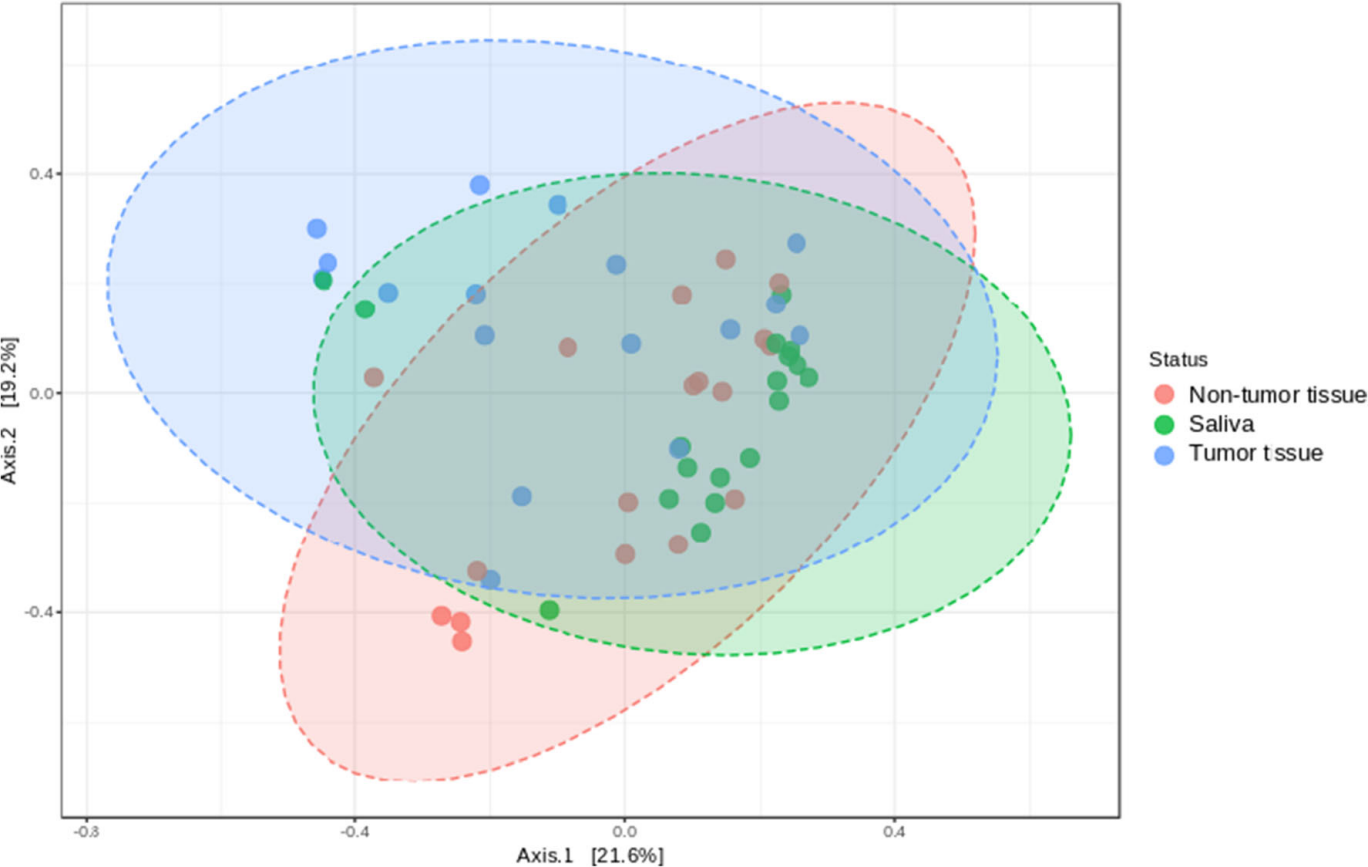
Principal coordinate analysis (PCoA) of samples based on the Bray-Curtis dissimilarity matrix. Each filled circle here represents a microbial community associated with non-tumor tissue (red), tumor tissue (blue), and saliva (green) with 95% confidence ellipses showing mean values of groups. A separation between groups was tested by comparing the principal coordinates using PERMANOVA statistical test (*p* value < 0.001). Further pairwise comparisons displayed significant differences in bacterial community composition among tumor and non-tumor (*p* = 0.00075) and saliva and tumor (*p* = 0.00075). Gaussian distribution of multivariate analysis of variance was tested to verify PERMANOVA assumptions (*p* value > 0.07)

**Fig. 2:**
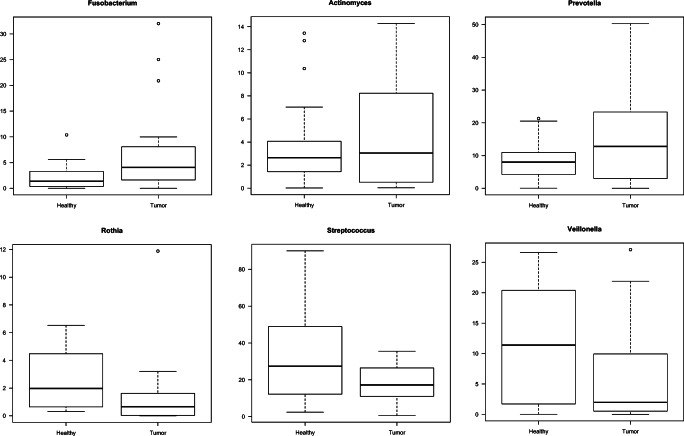
Box plots of the relative abundance of known oral pathogens and commensals. The top three box plots that indicate genera known to contain potentially pathogenic oral microbial species are enriched in TT, while the bottom three box plots that indicate genera commonly associated with oral health are in higher abundance in NT

**Fig. 3:**
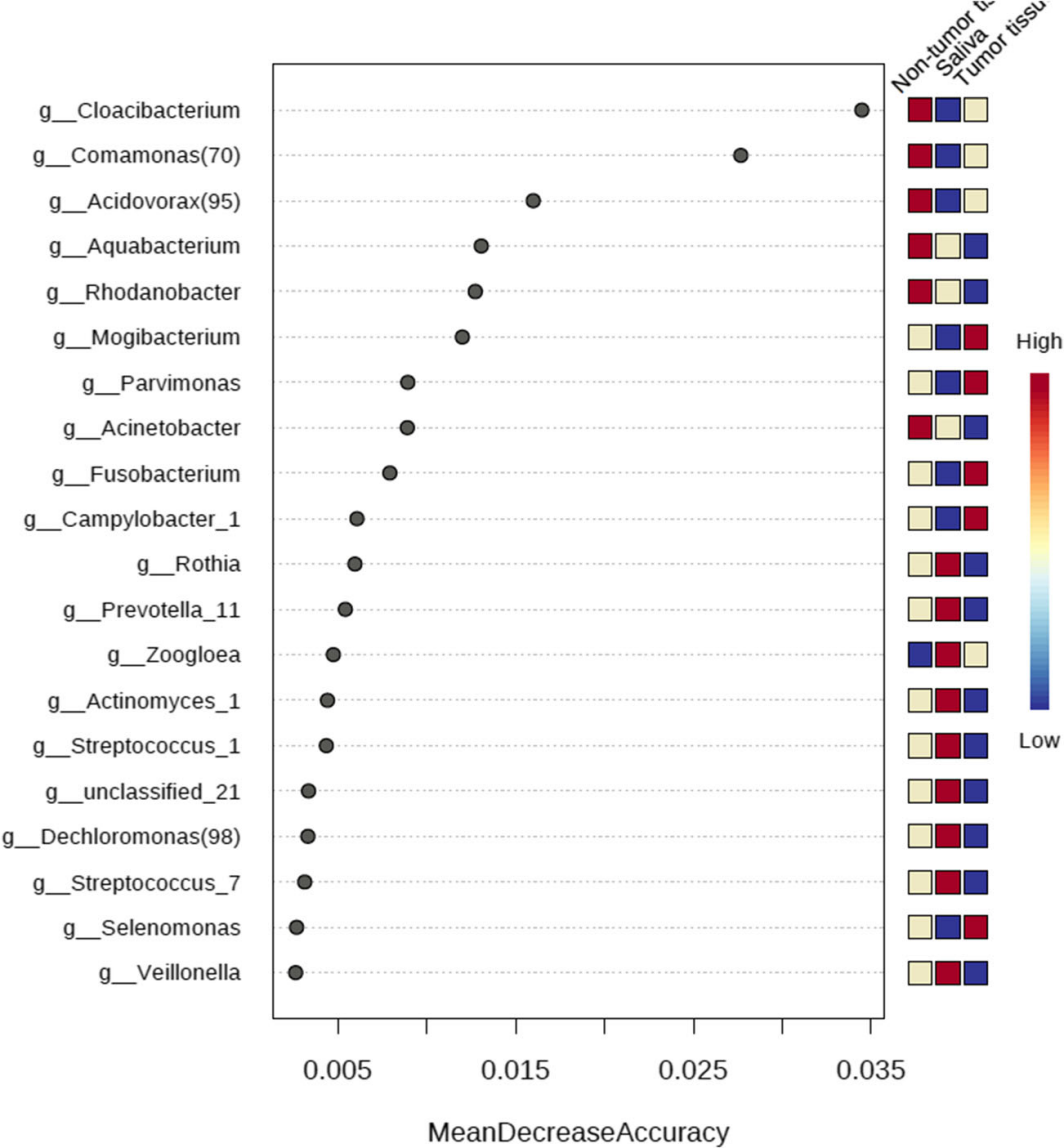
Random Forest classification model was applied on the 16S rRNA abundance data to identify most important microbial features in TT, NT, and saliva samples. Features with at least 4 reads and with a minimum prevalence of 10% across samples were included. Data was further transformed to centered log ratio (CLR) before applying the Random Forest classification algorithm

**Fig. 4:**
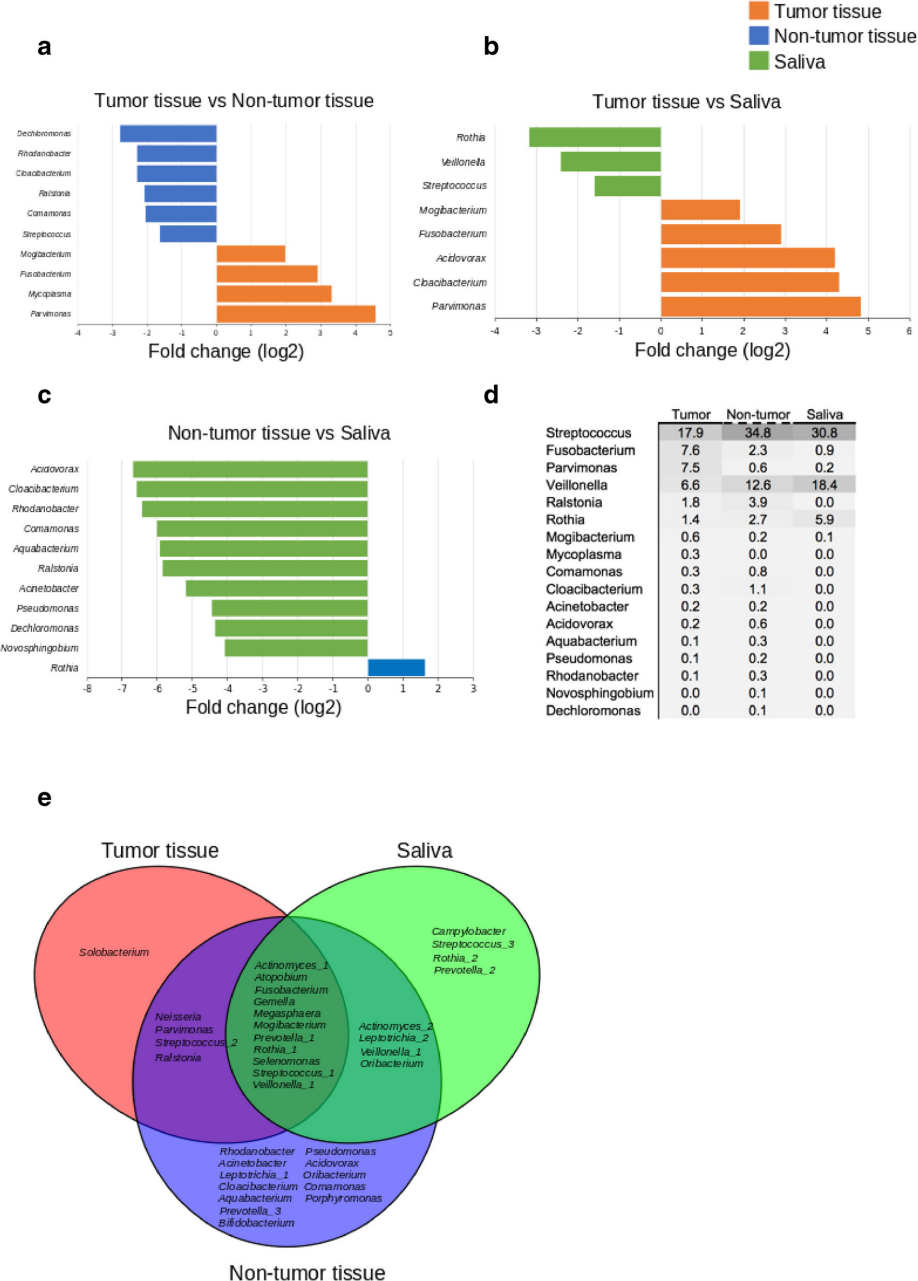
Differentially abundant taxa and core microbiome in the tumor, non-tumor, and saliva samples. Log2 fold changes of **a** tumor tissue vs non-tumor tissue, **b** tumor tissue vs saliva, and **c** non-tumor tissue vs saliva. **d** Heatmap presenting the relative abundances of differentially abundant genera. **e** A Venn diagram displaying core and unique genera represented at the OTU level (97% similarity)

The 16S sequence data showed that the microbial composition varied between TT and NT, while saliva samples mostly clustered with NT (Fig. 1). Using the adonis function in the vegan R package, we determined that the microbial composition associated with TT was significantly distinct when compared with contralateral NT (*p* = 0.001, *R*^2^ = 0.0757). SIMPER analysis [58] indicated that the top five discriminating genera between the TT and NT sample types included *Leptotrichia*, *Peptostreptococcus*, *Megasphaera*, *Rothia*, and one species of *Prevotella*. Other genera of interest included *Fusobacterium,* other species of *Prevotella*, and *Actinomyces*, which were in higher relative abundance in the TT when compared with the NT from OSCC samples (Fig. 2). Additionally, the sequence data showed that *Streptococcus*, *Veillonella*, and *Rothia* were higher in abundance in the NT than TT (Fig. 2). Random Forest analysis also indicated that TT was also shown to be highly abundant in *Fusobacterium*, *Parvimonas*, and *Mogibacterium* (Fig. 3). Sequencing analysis of saliva collected from matched TT and NT patient samples suggests that saliva samples appeared to be more similar to NT than TT (Fig. 1). Comparing the most abundant and core microbial taxa between the three sample types revealed three discernable communities in the oral cavity. Log2 fold changes in abundance of specific taxa was evident between the sample types (Fig. 4a–d). Taxa revealed to be in common between sample types as well as exclusive to each sample type is highlighted in Fig. 4e. Network analysis showed that co-occurrence between microbes within the sample types was revealed to be of high incidence in the TT and NT while low in saliva (Fig. 4e).

### Abundance of Oral Pathogens in Tumor Tissue and Non-Tumor Tissue

**Fig. 5:**
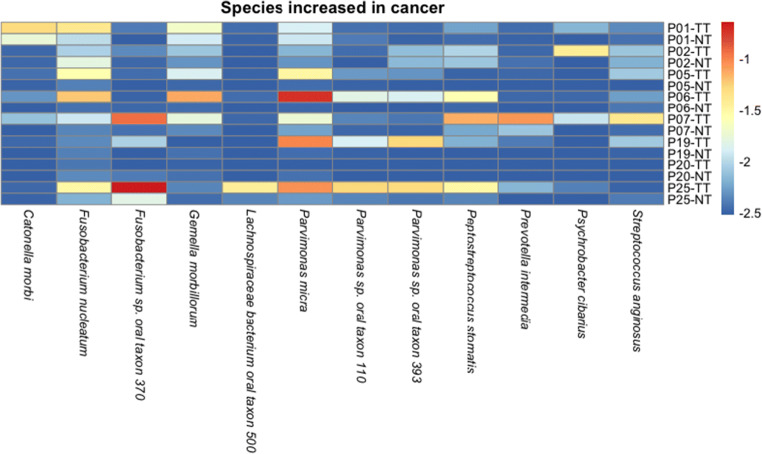
Heatmap of microbial abundance in tumor and non-tumor tissues. Species shown on the x-axis in this heatmap are demonstrated to be in higher abundance in tumor samples when compared with non-tumor samples. The individual patient IDs are listed followed by a TT or NT, indicating tumor tissue or non-tumor tissue, respectively

Analysis of 16S and metagenomic data revealed a relative abundance of microbial and fungal communities in all three sample types that illustrate a higher abundance of known oral pathogens exclusive to TT (Figure S1).TT microbial composition was higher in the abundance of known and opportunistic oral pathogens while having a decreased amount of known oral commensal bacteria when compared with NT. Taxonomic analysis revealed that a substantial percentage of sequence data belonging to genera known to contain oral pathogens or opportunistic oral pathogens such as *Prevotella* (17%), *Streptococcus* (17%), *Parvimonas* (8%), *Fusobacterium* (8%), *Peptostreptococcus* (2%), and *Porphyromonas* (2%) was present in TT. Our metagenomics data indicated that fusobacterial species in high abundance in TT included *Fusobacterium sp.* oral taxon 370 and *F. nucleatum*. *F. periodonticum* and *F. necrophorum* also appeared to be in high abundance in patients 25 and 7, respectively, while three patients, 7, 1, and 6, had higher levels of *Prevotella* species present in TT (Fig. 5). Additionally, the increased presence of *Parvimonas micra* in 63% of TTs suggests an exclusive association with OSCC (Fig. 5). Our data also showed that the percentage of sequence data belonging to genera known to contain oral commensal bacteria such as *Streptococcus* (33%) and *Veillonella* (12%) was higher in NT. Additionally, oral pathogens and opportunistic pathogens observed in TT are demonstrated to be in much lower abundance in NT, inclusive of *Parvimonas* (0.7%), *Fusobacterium* (2%), *Peptostreptococcus* (0.4%), and *Porphyromonas* (0.6%).

### Functional Potential and Association of Virulence Factors with OSCC Tissue

Analysis of functional potential of metagenome sequences showed 35 distinct BGCs, which were significantly abundant in TT samples when compared with NT samples. Relative increased abundance from the TT and NT samples are highlighted in blue and yellow, respectively (Table 1). Metagenomic sequencing also revealed several virulence factors that were significantly abundant in TT when compared with NT (Table 2). Open reading frames from our dataset searched against the VFDB revealed 9326 virulence homologs covering the broad diversity of 304 oral bacterial taxa (potential oral virulence gene sequences and their corresponding annotations are provided in the supplementary information). Additionally, we identified 3584 homologs with sequence identity > 75% and with a coverage of sequence length > 75% (potential ARG sequences and the annotations are provided in the supplementary information). Gene annotation showed that these virulence factors included enzymes with various functions, such as metabolic regulators, and extracellular surface protein structures. Two-way ANOVA testing showed that *Streptococcus pneumoniae* choline binding protein, *Haemophilus somnus* glycosyl transferases, along with *Acinetobacter baumannii* transcriptional regulator LysR, were enriched in TT; *p* values = 0.0291, 0.0004, and < 0.0001, respectively. Notably, three variants of fibronectin binding proteins were shown to be significantly abundant in TT. Two-way ANOVA testing showed that *S. gordonii* CshA and CshB fibronectin binding proteins were significantly higher in abundance in TT; *p* values = 0.0050 and 0.0199, respectively.

**Table 1:**
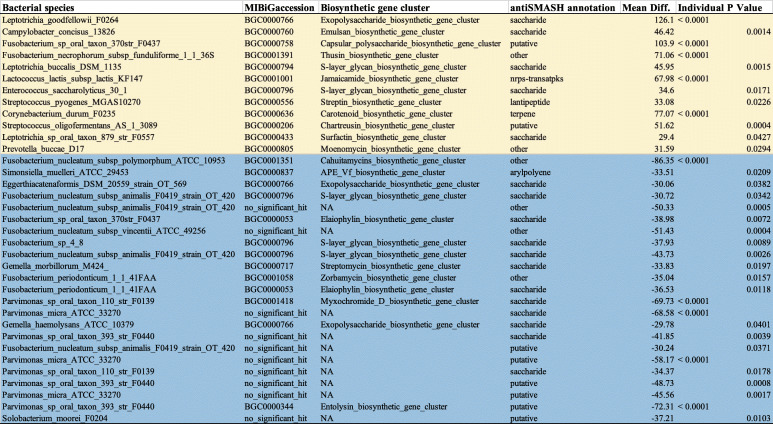
Biosynthetic gene clusters (BGCs) reveal microbial factors associated with OSCC. List of BGCs and associated bacterial species and significance values. Rows highlighted in yellow indicate BGCs that are higher in abundance in NT, while rows highlighted in blue indicate BGCs that are higher in abundance in TT

**Table 2:** Microbial virulence factor abundance in TT. Virulence factors found to be in higher abundance in TT when compared with contralateral NT samples. Predicted function, associated microbial taxa, and significance values are summarized below

Significantly abundant virulence factors in tumor tissue		
Taxa match	Gene annotation	Individual *p* value
*Streptococcus pneumoniae* Hungary 19A-6	Choline binding protein CVF122	0.0291
Treponema denticola ATCC35405	Major outer sheath protein (Fibronectin binding protein AI239)	< 0.0001
Mycoplasma penetrans HF-2	p35 lipoprotein homolog	0.0005
Acinetobacter baumannii AB0057	AB57 0984. transcriptional regulator, LysR (Heme utilization)	< 0.0001
Shigella dysenteriae Sd197	ospC4 Mxi-Spa TTSS effector controlled by VirB CVF494	0.0169
*Bacillus cereus* ATCC 10987	O-antigen polymerase (wzy) Polysaccharide capsule CVF567	0.0013
Haemophilus somnus 2336	lic2A glycosyl transferase family 25 LOS CVF494	0.0004
Streptococcus gordonii str. Challis	csh B surface-associated protein Csh B Fibronectin binding protein AI195	0.0199
Streptococcus gordonii str. Challis	csh A surface-associated protein Csh B Fibronectin binding protein AI194	0.0050

### Relevance of Fusobacterium Virulence Factors and Antibiotic Resistance Functions in Non-Tumor and Tumor Tissue–Associated Metagenomes

**Fig. 6:**
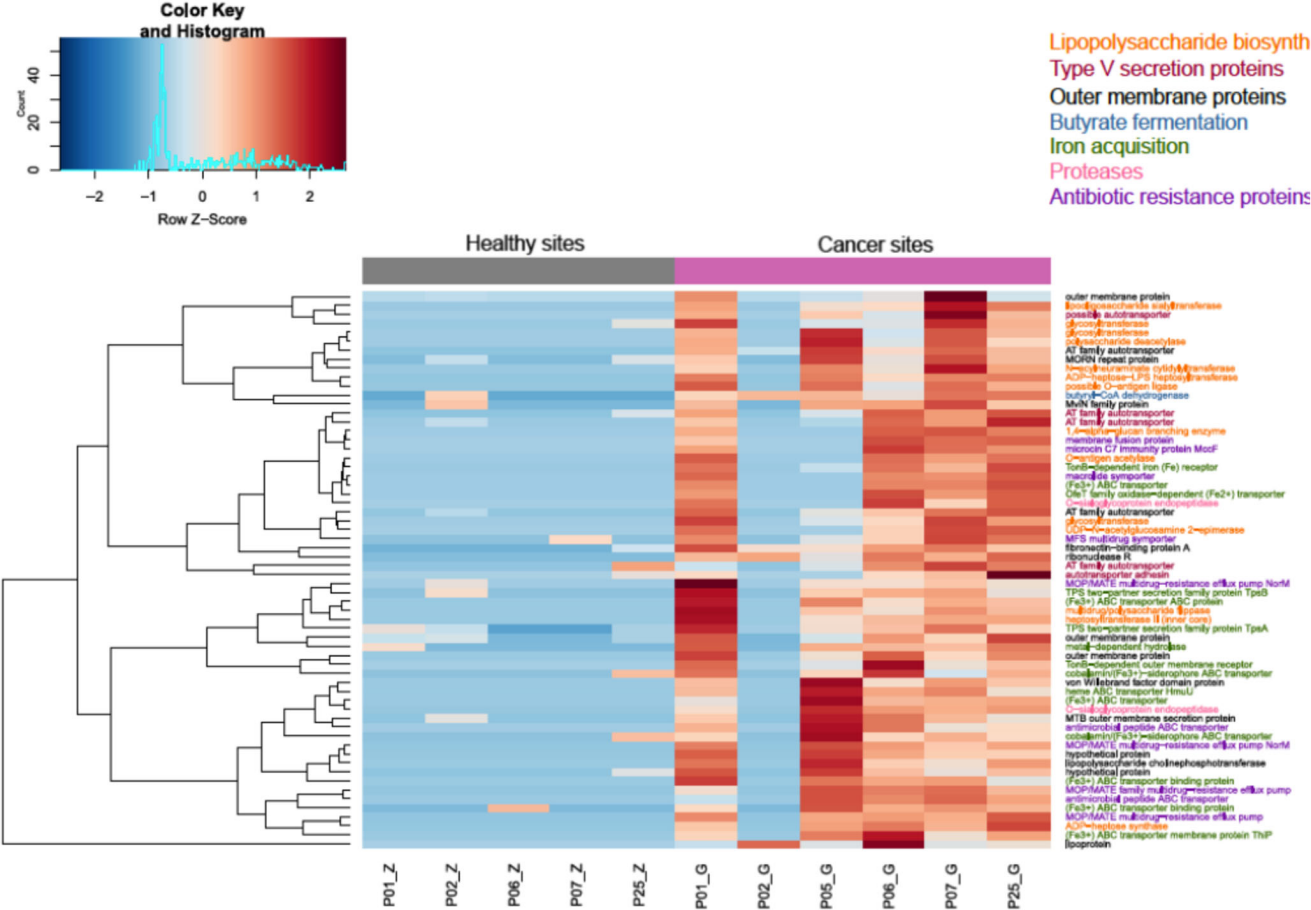
Clustered heatmap of Pearson-correlation coefficient for significantly enriched virulence and antibiotic resistance genes from *Fusobacterium*, representing non-tumor tissue and tumor tissue metagenomes. Clustering based on the DESeq2 generated normalized abundances for these genes clearly illustrates their associations with cancer

Among the 31,729 reads that mapped to *Fusobacterium* virulence factors and antibiotic resistance genes, a substantial fraction of which, 99.48% (31,564 reads), mapped to cancer metagenomes and only 0.5% (165 reads) of these sequences mapped to non-cancer metagenomes. In total, we identified 62 genes that were differentially enriched in OSCC metagenomes as compared with healthy samples highlighting their pathogenic potential (Fig. 6). These genes displayed features of adhesion, secretion, transport, resistance, and invasion. Genes encoding outer membrane proteins such as bacterial autotransporters, potential adhesion proteins including a fibronectin-binding protein, a possible autotransporter adhesion, and von Willebrand proteins were highly abundant in OSCC metagenomes. In particular, we identified a high abundance of MORN2 sequence repeats, which have been recently associated with actively invading *F. nucleatum* species [59]. In addition, we identified Type V secretion system encompassing autotransporter and two-partner secretion system genes. Also, genes associated with host immune evasion such as lipooligosaccharide sialyltransferase, which incorporates sialic acid into lipopolysaccharide biosynthesis, were more abundant in TT than NT. Additionally, endotoxin-related lipopolysaccharide biosynthetic genes were enriched in TT. Genes involved in iron acquisition such as iron ABC transporters, various iron receptors, and other siderophores were significantly in higher abundance in OSCC metagenomes. With respect to antibiotic resistance functions, we identified a high abundance of several antibiotic transporters including MOP/MATE family multidrug efflux pumps and drug/metabolite transporters. Lastly, we identified a high abundance of genes associated with lipopolysaccharide production such as glycosyl transferases, flippases, and deacetylases in TT.

### OSCC Patient Saliva Revealed Higher Concentrations of Microbial Proteins

Metaproteomics analysis resulted in the identification of 2300 protein groups (FDR < 1%) from the five OSCC saliva samples, including 1132 human proteins and 1168 from bacteria. Meanwhile, to investigate any OSCC-specific metaproteome profile, we analyzed in-parallel another five non-OSCC derived saliva samples from a separate ongoing project. These five non-OSCC samples served as a control. The number of identified unique protein groups, as well as the number of peptide-spectrum matches (PSMs), was plotted across the 10 samples (Figure S2). On average, 294 and 78 microbial proteins with corresponding 2122 and 543 PSMs were identified from OSCC saliva and control saliva samples, respectively, which contributed to 2.1% and 6.9% of total protein mass. From one particular sample (O9), around 51% of the identified protein groups were from oral bacteria representing 79 distinct genera, suggesting a high diversity of the oral microbiota obtained by metaproteomics approach (Figure S3). However, human proteins constituted almost 81% of the total protein intensities measured in this saliva sample, indicating a possible association of saliva microbiome with abundant host proteins. In another OSCC saliva sample (O36), a significant number of microbial proteins (450) were also identified, constituting 10% of total protein mass. These data have clearly shown the in-depth coverage of saliva metaproteome by the MS-based approach. In the context of microbial diversity, *Prevotella* appeared to be the most abundant genus identified from OSCC saliva, and up to 56% of the oral microorganisms were from the genus *Prevotella* (30.7 ± 17.9%, *n* = 5). This organism showed significantly (*p* < 0.05) higher abundance in the OSCC saliva when compared with non-OSCC saliva (Figure S2). Other organisms such as *Corynebacterium*, *Enterococcus*, and *Mogibacterium* were shown to have significantly decreased abundance in OSCC saliva when compared with non-OSCC saliva (Figure S2).

## Discussion

Mucosal homeostasis between the human microbiome and the host is the key to reducing inflammation and maintaining healthy host tissue. Virulence factors from pathobionts and pathogenic biofilms, such as those formed by *Fusobacterium*, disrupt tissue homeostasis leading to dysbiosis and disease development [60–62]. Uncontrolled mucosal tissue response is a hallmark of oral diseases, including gingivitis, mucositis, periodontitis, endodontic lesions, leukoplakia, and OC. Early stage OC, such as OSCC, has a relatively good prognosis, yet the majority of clinical cases are still diagnosed in advanced stages of the disease with a significant impact on patient prognosis and patient survival. Hence, there is an urgent need for understanding the role of oral pathobionts in OSCC development, thus facilitating the discovery of novel microbial and biofilm biomarkers dictating dysbiosis through the use of multi-omics approaches as done in our study, ultimately leading to the development of molecular diagnostics to improve early detection of OSCC and potentially novel therapeutics.

In the current study, we address the challenges associated with diagnostics of OSCC and obtained tissue samples to uncover microbial differences in tumor and non-tumor samples from each patient. We were able to discriminate our TT and NT samples by using SIMPER and Random Forest analysis to determine the variation in relative abundance. Our SIMPER analysis results indicated that one of the top five discriminating taxa that is the most abundant in TT when compared with NT, *Leptotrichia*, may potentially contribute to pathogenesis as this genus contains a number of pathogenic species such as *Leptotrichia hofstadii* and *L. stadii*, which have been isolated from oral wounds [63]. Additionally, as a Gram-negative organism, it is likely contributing to a host inflammatory response due to the characteristic lipopolysaccharide cell wall. Review of taxonomic relative abundance also demonstrated a higher proportion of oral genera known to contain pathogenic species in TT when compared with NT. This is particularly of interest as oral pathogens such as *Fusobacterium* have been previously associated with certain cancers [64, 65] and are observed to be in higher abundance in our TT samples emphasizing the significance of this finding in OSCC. Additionally, invasive pathogens such as *Fusobacterium* can be detected in our oral tissue samples while previous studies utilized superficial swabbing sampling techniques, which may not capture invasive oral pathogens associated with OSCC. Random forest analysis supported the results demonstrating higher proportions of Gram-negative organisms such as *Fusobacterium* and *Parvimonas* in TT.

The relative microbial compositions associated with tumor tissues presented significant differences when compared with contralateral non-tumor tissues (*p* = 0.001, *R*^2^ = 0.0757). Our results indicated that specific genera known to contain pathogenic and potentially pathogenic oral microbial species and strains such as *Fusobacterium, Prevotella,* and *Actinomyces* were detected in high abundance in tumor tissues. The potential pathogenicity of many species in each genera is of significance since oral pathogens such as *Prevotella intermedia* and *Prevotella nigrescens* has been implicated in contributing to periodontal disease [66–68]. Additionally, since our TT sequence data showed presence of *Porphyromonas gingivalis*, a known oral pathogen associated with periodontal disease and more recently OSCC [69], the known association of *P. intermedia* with *P. gingivalis* [68] provides additional evidence that these pathogens may be contributing to OSCC pathogenesis as periodontal disease is often associated with OC and other cancers [70–72]. Expanding on this study to include deeper metagenomics sequencing to confirm the presence of these species would certainly support our findings. Contrary to the increased abundance of specific genera known to contain oral pathogens associated within tumor tissues, we have also shown associations of several oral bacteria known to be found in healthy oral microbiomes. These genera including *Streptococcus, Veillonella,* and *Rothia* were observed in higher abundance in the NT samples when compared with TT samples. This finding is significant and further strengthens the assumption that OSCC pathology may be caused by oral pathogens.

The predominance and coexistence of the *Rothia* in the adjacent NT community, when compared with TT, were expected as *Rothia* species are part of the normal flora in the human oral cavity and pharynx [73, 74] and have also been isolated from the human gut microbiomes with moderate frequency [75, 76]. Additionally, *Rothia* is commonly found in homeostasis in oral communities of specific animal models including dogs [77]*.* The high abundance of *Streptococcus* in NT was also expected as this organism is also commonly seen in healthy oral microbiomes [28, 29]. Our results are consistent when compared with other studies that demonstrate that, in healthy humans, sequence analysis of oral communities showed the predominant taxa as *Corynebacterium*, *Rothia*, and *Actinomyces* [78] while the most prevalent species correlated with oxidative stress markers in an inter-individual specificity study were *Rothia* and *Streptococcus parasanguinis* [79].

Though our metagenomics sequencing was at shallow depth, we were able to detect several interesting findings. Our dataset showed TT community composition and suggested that there was coexistence in OSCC tissues, where *S. gordonii* and *Fusobacterium* genera were highly associated. Our metagenomics results showed that *S. gordonii* CshA and CshB fibronectin binding protein were significantly in higher abundance in TT; *p* values = 0.0050 and 0.0199, respectively, suggesting that association of *S. gordonii* to TT has some correlation to the development of OSCC. This association is significant since *S. gordonii* has been demonstrated as an early to mid-colonizer in oral biofilms [80–82] and may be suitable as a potential biomarker for diagnostics. Detecting increasing levels of *S. gordonii* fibronectin binding proteins over time in oral biopsy or deep swab samples may provide insight into a patient’s predisposition to the development of OSCC and could potentially revolutionize diagnostics of this disease. Additional experiments utilizing metagenomics as outlined in our study in addition to transcriptomics analysis will confirm our predictions and further support our results.

As inflammatory pathogens such as *Fusobacterium* [83, 84], *Porphyromonas* [84, 85], and *Parvimonas* [86] are likely to adhere to known commensal bacteria and, more importantly, to host epithelial cells, we justified the use of tissue samples in our study. Sample collections in regard to screening patients to determine their predisposition to OSCC would likely be improved if the collection of gum tissue biopsies or deep swabbing were utilized. We suspected that utilizing tissue samples over superficial swab samples as used in previous studies would provide a much more comprehensive description of community composition particularly invasive pathogens that may be deep in oral tissues. Two such invasive organisms, *Fusobacterium* and *Porphyromonas*, were frequently found in our data to be collectively in high abundance in TT. This is not entirely surprising since this pair of pathogens has also been isolated simultaneously in other periodontal studies in animal models [87] and human infections [86, 88]. These studies indicated enhanced pathogenicity of the mixed inocula in comparison with the individual confrontation. It is highly likely that frequency and natural coexistence of specific pathogens as demonstrated in our study may contribute to microbial synergism and virulence.

After evaluation of our sequencing data, we identified a number of potential biomarkers using various genomics approaches. However, since our metagenomics sequencing coverage was shallow due to host DNA contamination, we further evaluated the role of other microbial virulence factors in TT compared with the adjacent NT using predicted functional approaches to analyze the 16S sequences. Tax4fun analysis demonstrated predicted functional factors from microbial communities based on our 16S rRNA sequences, indicating that nucleotide metabolism, terpenoid and polyketide biosynthesis, cofactors and vitamins, and carbohydrate metabolism were enriched in TT, providing additional potential biomarkers to target to improve OSCC diagnostics. While most dynamics of virulence factors are still unknown in complex communities, it is probable that they are contributing to the survival of pathogenic bacteria associated with OSCC, immune evasion, inflammation, and disease progression.

Our results provide significant insight into OSCC pathology due to evidence and association of *Fusobacteria* with TT samples. *Fusobacteria* have long been associated with colon cancer [15, 36] and other types of cancers [37], and their pathogenic phenotype is classified as oncobacterium [89]. We found that significantly enriched virulence factors and antibiotic resistance genes from potentially oncogenic *Fusobacterium* are present in tumor metagenomes. Lipopolysaccharide biosynthesis, type V secretion proteins, outer membrane proteins, butyrate fermentation, and iron metabolism were further investigated. While butyrate production has been associated with suppression of inflammation and cancer progression, recent studies suggest that butyrate may contribute to cancer progression as pathogenic *Fusobacterium* utilizes a distinct amino acid metabolism pathway associated with the release of harmful by-products such as ammonia [90]. Clustering based on the DESeq2 generated normalized abundances for these genes illustrated their associations with OSCC metagenomes, especially outer membrane proteins and iron metabolism (Fig. 6). This illustrates the presence, abundance, and metabolic functions of Fusobacteria in inflammatory processes and dysbiosis and agrees with other studies that have been performed to date on OSCC.

In addition to exploring bacterial metagenomes obtained from tissue samples, we have also validated our findings through saliva analysis. Krona plots produced radial space-filling charts displaying the mean relative abundances of bacterial taxa based on 16S gene sequencing, as well as bacterial and fungal taxa based on the metagenomic sequencing. Figure S1 displays taxonomic hierarchy with genus and species levels at the outermost circle, characterized using 16S sequencing and metagenomics. An interactive version of these charts is available in Supplementary Figure S1. Our saliva results appeared to resemble the non-tumor tissue, as evidenced by Krona and PCoA plots (Fig. 1; Figure S1). Beyond deep sequencing of DNA, we utilized other approaches such as mass spectrometry (MS)–based metaproteomics as these methods have emerged as a complementary tool to transcriptomic analysis, allowing for simultaneous measurement of proteins derived from the host and microbiome. Since we have previously identified several human salivary proteins that have high prediction accuracy as biomarkers for OSCC [14], we further characterized the metaproteomic composition of the same set of saliva samples by taking advantage of the robust STrap sampling approach [14]. This method utilizes MS with high resolution accurate mass (HR/AM) capacity and with our previous metaproteomics experience [91] to study the host and microbial composition of saliva derived from OSCC patients. Our data demonstrated the significantly increased abundance of *Prevotella* in OSCC saliva when compared with non-OSCC saliva, thus validating our sequencing data while in agreement with previous findings [92, 93]. Our proteomics results indicated that specific bacteria such as *Corynebacterium*, *Enterococcus*, and *Mogibacterium* were mostly associated with non-OSCC saliva samples and in significantly decreased abundance in the OSCC saliva samples. *Corynebacterium* and other medically relevant Gram-positive rods have been found to be present in healthy plaque samples in addition to other parts of the body including nasal tissues [94], skin, and gut [95]. Such differentiations were not observed from the sequence data of the tissue samples, which showed that saliva was more similar to NT than TT. Our results further suggested that saliva may not reflect the microbial community of TT and is unlikely to be a suitable target specimen for OSCC diagnostics when using sequencing only. Proteomics results, however, suggest that this approach may be critical for the improvement of diagnostics and would provide a sample target that is less invasive than using deep swabbing or gum tissue biopsies. These data highlighted the importance of sampling sublocation for microbiome-associated biomarker discovery [96]. Combining multi-omics approaches with multiple sample sources would better elucidate disease signatures. In the context of total microbial mass in saliva metaproteome, although greater numbers of microbial proteins were identified in OCSS-derived saliva, as evidenced by the normalized spectrum counts, the statistical significance was low (*p* > 0.05) between OSCC and control subjects due to the small sample size. The key finding in our results is that the microbial diversity in OSCC saliva appears to have a higher abundance of microbial peptides when compared with non-OSCC saliva. We anticipate that analyzing a larger cohort would confirm our findings and reveal additional microbial markers.

Multi-omic approaches have been adapted to study the functional composition of the microbial community, each of which has strengths and weaknesses [97]. While our “genomic-proteomic” approach provided a comprehensive approach to generating information in regard to microbial virulence in TT, future mechanistic studies are needed to investigate host dynamics to validate the exact role of virulence factors in OSSC pathogenesis. Altogether, our results showed that virulence factors from *S. gordonii* and *Fusobacterium* species were found mostly associated with TT when compared with NT and saliva. Though our use of tissues may not be practical in the clinical diagnostics environment, it was necessary to achieve a comprehensive characterization of the microbial community in OSCC. Additionally, we determined that saliva samples may provide clinicians with a less invasive approach to determining a patient’s predisposition to development of OSCC. We were also able to identify specific signatures that can be further utilized in diagnostics to determine a patient’s susceptibility to OSCC through analysis of tissue biopsies or perhaps aggressive oral tissue swabs. Expanding our study to generating data from patients at each stage of OSCC development is critical to fully understand the oral microbiome’s role in OSCC pathology. Furthermore, understanding the metabolic function of inflammatory virulence genes has great potential in diagnostics and treatment of OSCC as it could potentially shed light on their functions related to the host immune system to clear microbial biofilms and respond to the dysbiotic microbiome causing the disease state and/or the exacerbated host’s inflammatory response causing tissue collateral damage.

## Electronic Supplementary Material

ESM 1 (DOCX 518 kb)
