# Supplementary material for: Oral Microbial Species and Virulence Factors Associated with Oral Squamous Cell Carcinoma
Source: Microb Ecol. 2020 Nov 6;82(4):1030–46. doi: 10.1007/s00248-020-01596-5 (PMC8551143; doi:10.1007/s00248-020-01596-5)
Supplement: Supplementary file 1 — (DOCX 518 kb) [file 248_2020_1596_MOESM1_ESM.docx]

**Supplemental Figures: Oral Microbial Species and Virulence Factors Associated with Oral Squamous Cell Carcinoma.**

https://doi.org/10.1007/s00248-020-01596-5


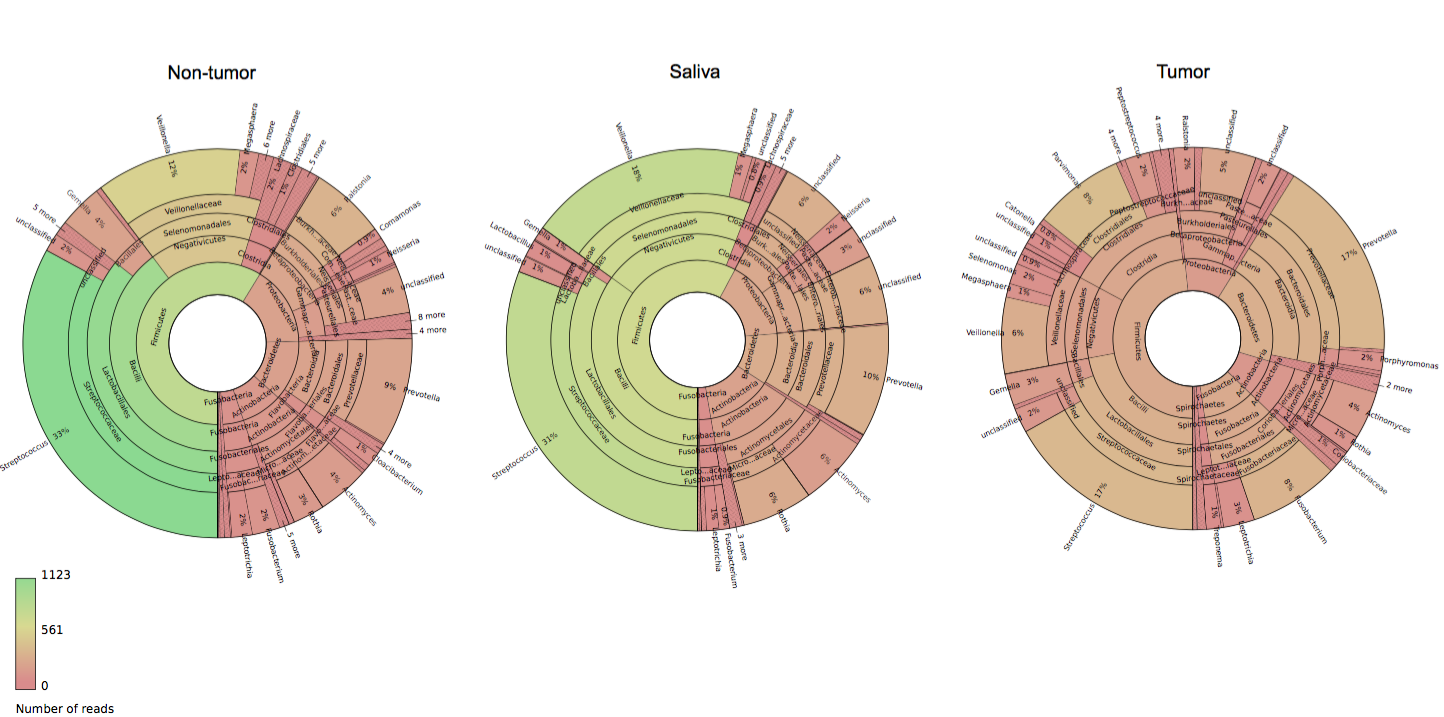


**Figure S1: Microbial composition in the tumor tissue, contralateral non-tumor tissue and saliva**.

Krona produced radial space-filling charts displaying the mean relative abundances of bacterial taxa based on 16S rRNA gene sequence. Circles display taxonomic hierarchy with genus and species levels at the outermost circle, characterized using 16S rRNA gene sequencing and metagenomics respectively. The average number of reads mapped to each taxon is colored from low (red) to high (green) indicating low and high abundant taxa. The interactive version of these charts is available in the supplementary figures.

**
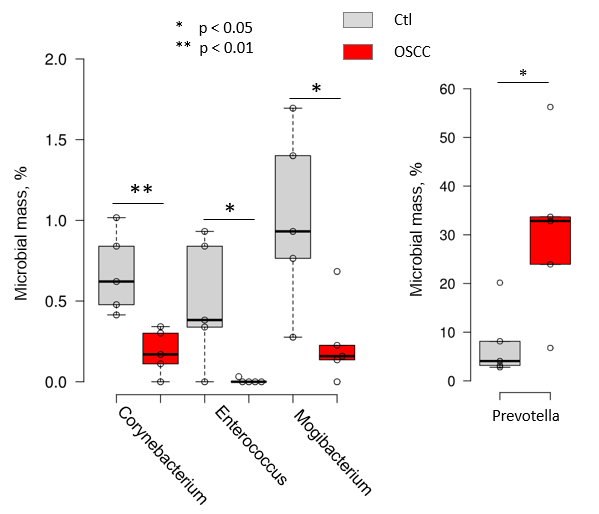
**

**Figure S2**: **Box plots of the relative abundance of saliva microorganisms revealed by metaproteomics.** The percent mass of differentially abundant microbial proteins highlighted between the OSCC and control samples.


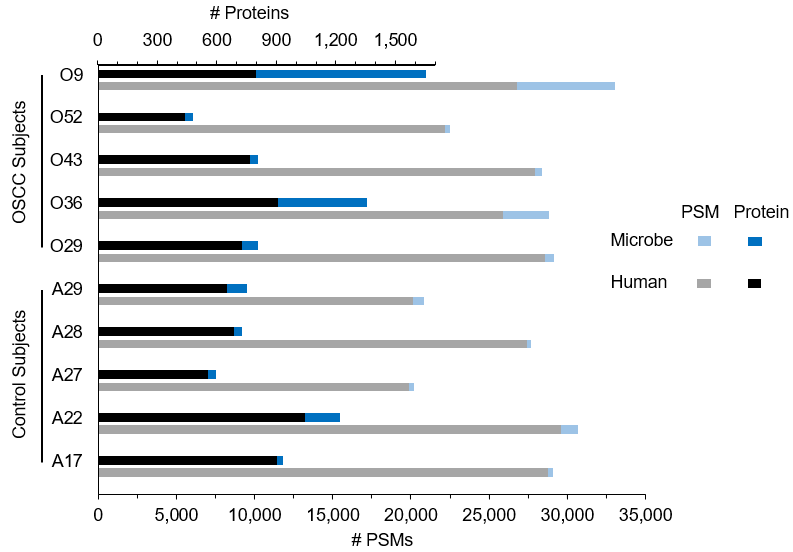


**Figure S3.** Metaproteome analyses of saliva samples. The number of protein identifications and peptide spectrum matches (PSMs) for the ten saliva samples were plotted.
